# Associations of combined physical activity and body mass index groups with colorectal cancer survival outcomes

**DOI:** 10.1186/s12885-023-10695-8

**Published:** 2023-04-03

**Authors:** Caroline Himbert, Jennifer Ose, Biljana Gigic, Richard Viskochil, Kelly Santuci, Tengda Lin, Anjelica Ashworth, Jessica N. Cohan, Courtney L. Scaife, Jolanta Jedrzkiewicz, Victoria Damerell, Katelyn M. Atkins, Jun Gong, Matthew G. Mutch, Corey Bernadt, Seth Felder, Julian Sanchez, Stacey A. Cohen, Mukta K. Krane, Nathan Hinkle, Elizabeth Wood, Anita R. Peoples, Jane C. Figueiredo, Adetunji T. Toriola, Erin M. Siegel, Christopher I. Li, David Shibata, Kenneth Boucher, June L. Round, Alexis B. Ulrich, Martin Schneider, Lyen C. Huang, Sheetal Hardikar, Cornelia M. Ulrich

**Affiliations:** 1 University of Utah, Salt Lake City, UT USA; 2 Huntsman Cancer Institute, Salt Lake City, UT USA; 3 Heidelberg University Hospital, Heidelberg, Germany; 4 University of Massachusetts Boston, Boston, MA USA; 5 Cedars-Sinai Medical Center, Los Angeles, CA USA; 6 Washington University School of Medicine, St. Louis, MO USA; 7 H. Lee Moffitt Cancer Center and Research Institute, Tampa, FL USA; 8 Fred Hutchinson Cancer Center, University of Washington, Seattle, WA USA; 9 University of Tennessee Health Science Center, Memphis, TN USA; 10 Rheinland Klinikum Neuss Lukas Krankenhaus, Neuss, Germany

**Keywords:** Colorectal cancer, Physical activity, Obesity, Energy balance, Survivorship

## Abstract

**Background:**

Physical activity and BMI have been individually associated with cancer survivorship but have not yet been studied in combinations in colorectal cancer patients. Here, we investigate individual and combined associations of physical activity and BMI groups with colorectal cancer survival outcomes.

**Methods:**

Self-reported physical activity levels (MET hrs/wk) were assessed using an adapted version of the International Physical Activity Questionnaire (IPAQ) at baseline in 931 patients with stage I-III colorectal cancer and classified into ‘highly active’ and’not-highly active’(≥ / < 18 MET hrs/wk). BMI (kg/m^2^) was categorized into ‘normal weight’, ‘overweight’, and ‘obese’. Patients were further classified into combined physical activity and BMI groups. Cox-proportional hazard models with Firth correction were computed to assess associations [hazard ratio (HR), 95% profile HR likelihood confidence interval (95% CI) between individual and combined physical activity and BMI groups with overall and disease-free survival in colorectal cancer patients.

**Results:**

‘Not-highly active’ compared to ‘highly active’ and ‘overweight’/ ‘obese’ compared to ‘normal weight’ patients had a 40–50% increased risk of death or recurrence (HR: 1.41 (95% CI: 0.99–2.06), *p* = 0.03; HR: 1.49 (95% CI: 1.02–2.21) and HR: 1.51 (95% CI: 1.02–2.26), *p* = 0.04, respectively). ‘Not-highly active’ patients had worse disease-free survival outcomes, regardless of their BMI, compared to ‘highly active/normal weight’ patients. ‘Not-highly active/obese’ patients had a 3.66 times increased risk of death or recurrence compared to ‘highly active/normal weight’ patients (HR: 4.66 (95% CI: 1.75–9.10), *p* = 0.002). Lower activity thresholds yielded smaller effect sizes.

**Conclusion:**

Physical activity and BMI were individually associated with disease-free survival among colorectal cancer patients. Physical activity seems to improve survival outcomes in patients regardless of their BMI.

**Supplementary Information:**

The online version contains supplementary material available at 10.1186/s12885-023-10695-8.

## Introduction

Components of energy balance including physical activity and body composition have individually been associated with colorectal cancer survival and recurrence [1–7]. Their combined effect on colorectal cancer outcomes has yet to be investigated [8]. The ‘fat-but-fit’ paradox, which refers to the observation that increasing physical activity levels can counteract the negative effects of obesity, has been mainly studied in the context of cardiometabolic health [8–11]. Improved cardiorespiratory fitness and reduced insulin resistance and overall metabolism are some of the beneficial effects through physical activity. On the molecular level, physical activity attenuates some of the obesity-induced changes including insulin resistance and chronic systemic inflammation [12, 13].

Both, physical activity and obesity have been presented as important risk factors throughout the cancer care continuum, particularly among breast and colon cancer survivors; they also predict clinical outcomes in cancer patients [1–7]. While the current physical activity guidelines from the American College of Sports Medicine (ACSM) for cancer survivors recommend at least 150 min of moderate to vigorous activity [≥ 8.75 metabolic equivalent per tasks (MET) hrs/wk] per week [14], higher activity thresholds of 15–18 MET hrs/wk have shown to be associated more strongly with improved survival among colorectal cancer patients [3, 5, 15–18]. While physical activity has been well studied in the context of survival, its effect on cancer recurrence remains unclear.

The relationship between BMI and colorectal cancer survival outcomes does not appear to be linear. Prior studies have demonstrated an obesity paradox, a J- or U-shaped relationship between BMI and colorectal cancer-specific and overall survival where patients who are overweight or class I obese have superior outcomes than patients who are underweight, normal weight, or class II and III obese [19–24]. Most studies have been conducted in stage I-III colorectal cancer patients [19–24]. In contrast, a positive linear relationship of BMI with recurrence risk among colorectal cancer patients has been previously observed [25, 26].

Here, we analyzed data from a large international cohort of stage I-III colorectal cancer patients to investigate individual and combined associations of physical activity and BMI groups with overall and disease-free survival in colorectal cancer patients. In separate analyses, we tested two clinically-indicated physical activity thresholds: 1) < / ≥ 18 MET hrs/wk (~ 90 min of jogging or 6 h of walking per week) based on previous research that has identified this threshold to improve survival specifically in colorectal cancer patients [5, 16, 17], and 2) < / ≥ 8.75 MET hrs/wk according to the current physical activity guidelines for cancer survivors [14, 27]. Results from this study will contribute to our understanding of link between energy balance and outcomes in colorectal cancer survivorship.

## Methods

### Study population

The present study is conducted as part of the prospective, multicenter ColoCare Study (ClinicalTrials.gov NCT02328677, first trial registration: 31/12/2014), an international cohort of newly diagnosed stage I–IV colorectal cancer patients (ICD-10 C18–C20) [28]. The ColoCare Study design has previously been described [28–30]. Briefly, the ColoCare Study is a multicenter cohort of transdisciplinary research on colorectal cancer outcomes and prognosis, with the following inclusion criteria: patients first diagnosed with colon or rectal cancer (stages I–IV), age > 18 year, English (US sites) or German (German site) speaking, and mentally/physically able to consent and participate. Participants were staged according to the American Joint Committee on Cancer (AJCC) 8^th^ edition system based on histopathologic findings. All analyses in this manuscript are based on data collected from 931 patients with stage I-III colorectal cancer enrolled between October 2010 and August 2021 at the ColoCare Study sites in the United States (US) at the Fred Hutchinson Cancer Research Center (Seattle, WA), the Huntsman Cancer Institute (Salt Lake City, UT), Cedars-Sinai Samuel Oschin Comprehensive Cancer Center (Los Angeles, CA), and Washington University in St. Louis (St. Louis, MO), and in Germany at the Cancer National Center for Tumor Diseases (NCT) and University Hospital Heidelberg (Heidelberg, Germany) with available physical activity and BMI data at baseline. The study was approved by the institutional review boards of the respective institutions and all patients provided written informed consent.

### Exposure assessments

#### Physical activity assessment

Recreational physical activity within the past year before surgery was assessed at baseline (pre-surgery) using a self-administered adapted version of the International Physical Activity Questionnaire (IPAQ) questionnaire [31]. Multiple choice questions were used instead of the text entry questions in the original questionnaire version. Patients were asked if they walked for exercise, did moderate (e.g., leisure cycling) or vigorous (e.g., aerobics) exercise for at least 10 min over the past year (‘no’, ‘yes, less than once a week’, ‘yes, more than once a week’). If they responded ‘yes, more than once a week’, patients were asked about how many days per week (1–2, 3–4, 5–7), and how many minutes per day (10–29, 30–44, 45–59, 60 +). In addition, patients were asked about their usual walking pace (casual, moderate, fast). The IPAQ questionnaire captures data on usual recreational physical activity during the preceding year and has been previously validated [31, 32]. The summation of duration (hours) and frequency (per week) of physical activity can be calculated in metabolic equivalent tasks hours per week (MET hrs/wk) for each patient to determine the average amount of time per week that the patient spent in moderate to vigorous activity. The IPAQ scoring protocol was used for the computation of MET hrs/wk (available at: https://sites.google.com/site/theipaq/scoring-protocol). The assignment of MET values to activities follows the most recent Compendium of Physical Activities [33]. We excluded patients with missing data on physical activity (*n* = 392, 30%). Randomness of missing data was evaluated comparing baseline characteristics by those who had complete *vs.* those who had missing physical activity data. Further categorization of physical activity levels based on two different activity thresholds are explained in the statistical analyses section.

#### Body Mass Index (BMI)

BMI (kg/m^2^) at baseline (pre-surgery) was abstracted from patient medical charts or anesthesia protocols. We conducted a blinded review of a subset of charts (10%, *n* = 250) across sites to ensure quality of the data abstraction. Patients who were underweight (BMI ≤ 18 kg/m^2^, n = 26) were excluded from the analyses given that the majority (77%) was advanced stage disease (stages III-IV).

### Survival outcomes

Medical chart abstraction, as well as linkages to cancer registry and vital status records were used to obtain detailed information on clinical outcomes (e.g., survival, recurrence) in the ColoCare Study [28, 34, 35]. Survival was ascertained through reviews of patient medical records, state or national cancer and death registries at repeat intervals after surgery. Recurrence was ascertained using medical records, including pathology reports after surgery.

Overall survival was defined as the time a patient survives from surgery until death or last observation (August 2021). Disease-free survival was defined as the time a patient survived from surgery date until either disease progression, death, or last observation. Risk of recurrence was defined as the time from the surgery date until local or metastatic recurrence.

Person-years were calculated from the surgery date until the date of death (overall survival), date of death or recurrence (disease-free survival). Non-fixed censoring was applied and patients with any follow-up time were included to maximize study power. Patients who died within 30 days after surgery (*n* = 7) were excluded from all analyses given the cause of death was likely related to surgical complications. Similarly, patients who had a recurrence within 30 days after surgery (*n* = 2) were excluded from all analyses given that these patients were likely never disease free.

### Statistical analyses

#### Combined physical activity and BMI groups

Moderate activity was defined as 3.5 to 6 MET hrs and vigorous activities as ≥ 6 MET hrs [36]. Patients were categorized based on two different physical activity thresholds into: 1) ‘highly active’ and ‘not-highly active’ (< / ≥ 18 MET hrs/wk) based on previous research that has identified this threshold to improve survival specifically in colorectal cancer patients [5, 16, 17], and 2) ‘active’ and ‘inactive’ (< / ≥ 8.75 MET hrs/wk) according to the current physical activity guidelines for cancer survivors [14, 27]. For sensitivity analyses, physical activity levels were categorized into quartiles to test a dose–response relationship between physical activity and survival outcomes. Patients were categorized into ‘normal weight’ (BMI: < 25 kg/m^2^), ‘overweight’ (BMI: ≥ 25 and < 30 kg/m^2^), and ‘obese’ (BMI: ≥ 30 kg/m^2^) [37]. Combining physical activity and BMI information, patients were further categorized into (see Supplementary Figure S1):

- 1) ‘highly active or active/normal weight’ (≥ 18 or ≥ 8.75 MET hrs/wk, < 25 kg/m^2^),
- 2) ‘not-highly active or inactive/normal weight’ (< 18 or < 8.75 MET hrs/wk, < 25 kg/m^2^),
- 3) ‘highly active or active/overweight’ (≥ 18 or ≥ 8.75 MET hrs/wk, ≥ 25 and < 30 kg/m^2^),
- 4) ‘not-highly active or inactive/overweight’ (<18 or <8.75 MET hrs/wk, ≥25 and <30 kg/m^2^),
- 5) ‘highly active or active/obese’ (≥ 18 or ≥ 8.75 MET hrs/wk, ≥ 30 kg/m^2^), and
- 6) ‘not-highly active or inactive/obese’ (< 18 or < 8.75 MET hrs/wk, ≥ 30 kg/m^2^).

#### Statistical analyses

Mean values and proportions for continuous variables as well as frequencies and percentages for categorical variables to describe patient characteristics at baseline were computed. Cox-proportional hazard models were used to estimate hazard ratios (HR) and 95% profile HR likelihood confidence intervals (CI) for overall and disease-free survival. Firth correction was added to the models to account for limited power. Time zero was the date of surgery and concluded by the date of death (or recurrence), or last observation, whichever came first. Computation and visualization of scaled Shoenfield residuals against time were used to test the proportionality of hazards assumption for all included independent variables [38, 39]. Martingale Residuals were plotted to test for non-linearity. Significance tests for differences in survival curves across BMI/physical activity categories were assessed using the Wald χ^2^ test and *p* < 0.05 was deemed statistically significant. Stratified analyses were conducted to identify effect modification. Sex (male, female) and tumor site (colon, rectum) were tested for effect modification. The following variables were evaluated for potential confounding: sex (male, female), age (< 50, ≥ 50 years), race (White, non-White), tumor stage (I, II, and III) and site (colon, rectum), neo-adjuvant treatment (yes, no),adjuvant treatment (yes, no), and smoking (never, former, current). Models were adjusted for potential confounders. Robustness of the model and confounding effects of relevant factors were assessed using standard methods, including an evaluation whether risk estimates changed > 10% after inclusion of a covariate and likelihood ratio test. Bonferroni correction was used to correct adjusted *p-*values for multiple testing. The final model included sex, age, tumor stage, and adjuvant treatment. All statistical analyses were performed in SAS analytics software.

## Results

Table 1 summarizes baseline characteristics of the study population by combined physical activity (8.75 MET hrs/wk) and BMI categories. *N* = 931 patients were included in the analyses. Patients were on average 60 years old and 43% were female. Patients who were classified as ‘active’ were on average under 60 years old regardless of their BMI and ‘inactive’ patients were more likely over the age of 60 years. A greater proportion of men were ‘overweight’ or ‘obese’ compared to women regardless of their physical activity levels. More than half of the study population was diagnosed with colon cancer (51%) and stages II and III (28% and 49%, respectively). The highest proportion of colon cancer cases was observed among ‘obese/inactive’ patients. Thirty percent underwent neoadjuvant and 43% underwent adjuvant treatment. The study population was distributed across combined physical activity and BMI groups as follows: *n* = 142 ‘active/normal weight’, *n* = 125 ‘inactive/normal weight’, *n* = 166 ‘active/overweight’, *n* = 187 ‘inactive/overweight’, *n* = 99 ‘active/obese’, and *n* = 212 ‘inactive/obese’.

**Table 1:** Baseline study population characteristics by combined physical activity (8.75 MET hrs/wk) and BMI groups [n (%) if not otherwise stated]

	**Normal weight**		**Overweight**		**Obese**		**Total**
	**Active**	**Inactive**	**Active**	**Inactive**	**Active**	**Inactive**	
**N**	142	125	166	187	99	212	931
**Age, mean (SD)**	57 (± 13)	62 (± 13)	59 (± 14)	62 (± 12)	59 (± 13)	62 (± 11)	60 (± 13)
**Sex**							
Female	77 (54)	64 (51)	58 (35)	71 (37)	30 (30)	105 (49)	405 (43)
Male	65 (46)	61 (49)	108 (65)	116 (63)	69 (70)	107 (51)	526 (57)
**Race**							
White	124 (87)	109 (88)	146 (88)	173 (93)	82 (83)	181 (86)	815 (87)
Non-White	18 (13)	14 (12)	20 (12)	14 (7)	17 (17)	27 (14)	110 (12)
**Ethnicity**							
Hispanic	2 (1)	4 (3)	4 (2)	4 (2)	2 (2)	13 (6)	29 (3)
Non-Hispanic	140 (99)	121 (97)	162 (98)	183 (98)	97 (98)	199 (94)	902 (97)
**Tumor stage**							
I	32 (23)	32 (26)	36 (22)	41 (22)	27 (27)	48 (23)	216 (23)
II	44 (31)	32 (26)	45 (27)	56 (30)	24 (24)	55 (26)	256 (28)
III	66 (47)	59 (48)	84 (51)	88 (47)	48 (49)	108 (51)	453 (49)
**Tumor site**							
Colon	73 (51)	59 (47)	82 (49)	93 (50)	50 (51)	121 (57)	478 (51)
Rectum	69 (49)	66 (53)	84 (51)	94 (50)	49 (49)	91 (43)	453 (49)
**Neoadjuvant treatment**							
Yes	44 (31)	44 (35)	60 (36)	55 (29)	27 (28)	49 (23)	279 (30)
No	98 (69)	81 (65)	106 (64)	132 (71)	72 (72)	163 (77)	652 (70)
**Adjuvant treatment**							
Yes	60 (42)	53 (42)	78 (47)	73 (39)	43 (43)	94 (44)	401 (43)
No	82 (58)	72 (58)	88 (53)	114 (61)	56 (57)	118 (56)	530 (57)

Not yet abstracted *n* = 6 for tumor stage. *N* = 6 missing for race

*SD*  – standard deviation, *MET hrs/wk* – metabolic equivalent per task in hours per week, *BMI* – body mass index

### Multivariable-adjusted models testing associations of individual physical activity and BMI groups with overall and disease-free survival among colorectal cancer patients

Table 2 summarizes the results testing associations of individual physical activity and BMI groups on overall and disease-free survival. As of August 2021, the median follow-up time was 34 months, *n* = 116 patients were deceased and *n* = 72 had experienced a recurrence. Associations between physical activity levels of 18 MET hrs/wk, but not 8.75 MET hrs/wk, with disease-free survival approached significance (*p* = 0.06, adjusted for multiple testing: *p* = 0.12). Patients reporting activity levels below the threshold of 18 MET hrs/wk had a marginally significantly increased risk of death or recurrence (41%) compared to those reporting levels above this threshold (*p* = 0.06, adjusted for multiple testing: *p* = 0.12). Analyses using physical activity levels categorized into quartiles showed a statistically significant trend between increased physical activity levels and improved survival outcomes (Supplementary Table S1). BMI groups were statistically significantly associated with disease-free survival. Both, ‘overweight’ and ‘obese’ patients had a 1.49 and 1.51-fold increased risk of death or recurrence compared to ‘normal weight’ patients (*p* = 0.04, respectively). No differences in results were observed when stratifying analyses by sex or tumor site (data not shown). Individual physical activity or BMI groups were not statistically significantly associated with overall survival.

**Table 2:** Cox proportional hazard models of individual physical activity and BMI groups with overall survival, and disease-free survival in colorectal cancer patients

	**Total N** **(N events)**	**HR (95% Profile HR Likelihood CI)**	***P*-value**	*P*-value*	**Type III *P*-value**
**Overall Survival**					
**Physical activity**					
**18 MET hrs/wk activity threshold**					
Highly active (≥ 18 MET hrs/wk)	216 (26)	1.00 (Ref)			
Not-highly active (< 18 MET hrs/wk)	599 (90)	1.27 (0.83–2.02)	0.29	0.50	–
**8.75 MET hrs/wk activity threshold**					
Active (≥ 8.75 MET hrs/wk)	362 (45)	1.00 (Ref)			
Inactive (< 8.75 MET hrs/wk)	453 (71)	1.31 (0.90–1.92)	0.16	0.29	–
**BMI**					
Normal weight (< 25 kg/m^2^)	242 (25)	1.00 (Ref)			
Overweight (≥ 25, < 30 kg/m^2^)	305 (48)	1.58 (0.98–2.62)	0.07	–	0.18
Obese (≥ 30 kg/m^2^)	268 (43)	1.48 (0.90–2.48)	0.14	–	
**Disease-free survival**					
**Physical activity**					
**18 MET hrs/wk activity threshold**					
Highly active (≥ 18 MET hrs/wk)	201 (38)	1.00 (Ref)			
Not-highly active (< 18 MET hrs/wk)	528 (150)	1.41 (0.99–2.06)	0.06	0.12	–
**8.75 MET hrs/wk activity threshold**					
Active (≥ 8.75 MET hrs/wk)	328 (74)	1.00 (Ref)			
Inactive (< 8.75 MET hrs/wk)	401 (114)	1.22 (0.90–1.64)	0.22	0.39	–
**BMI**					
Normal weight (< 25 kg/m^2^)	224 (40)	1.00 (Ref)			
Overweight (≥ 25, < 30 kg/m^2^)	270 (77)	**1.49 (1.02–2.21)**	**0.04**	**–**	0.08
Obese (≥ 30 kg/m^2^)	235 (71)	**1.51 (1.02–2.26)**	**0.04**	**–**	

^*^p value corrected for multiple testing using Bonferroni correction method. All analyses were adjusted for age, sex, tumor stage, adjuvant treatment, and mutually adjusted for baseline BMI (kg/m^2^) and physical activity (MET hrs/wk); *HR* – hazard ratio, *95% CI* – 95% – confidence interval, *MET* – metabolic equivalent per task, *hrs/wk* – hours/week; bold indicates statistical significance (*p* < 0.05)

### Multivariable-adjusted models testing associations of combined physical activity and BMI groups with overall and disease-free survival among colorectal cancer patients

**Fig. 1:**
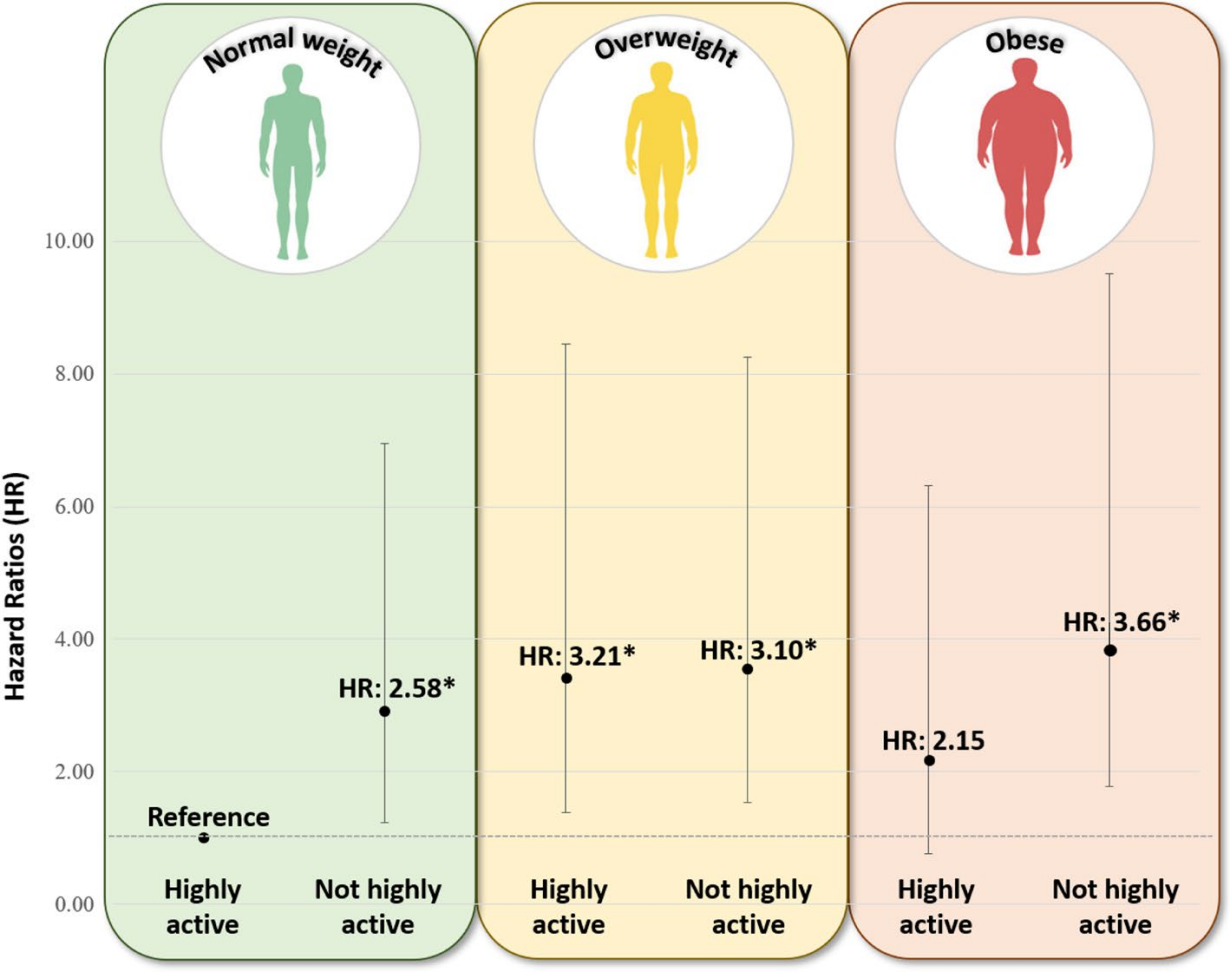
Associations of combined physical activity (18 MET hrs/wk threshold) and body mass index groups with disease-free survival in colorectal cancer patients. Data expressed in hazard ratios and 95% confidence intervals. *Indicates statistical significance (*p* < 0.05)

Table 3, Supplementary Table 1 and Fig. 1 illustrate results of associations of combined physical activity and BMI groups on overall and disease-free survival. We observed statistically significant associations between combined physical activity and BMI groups with overall and disease-free survival when using 18 MET hrs/wk as the activity threshold (Table 3 and Fig. 1). ‘Not-highly active/obese’ patients had a 2.43-fold increased risk of dying compared to ‘active/normal weight’ patients (*p* = 0.05, adjusted for multiple testing: *p* = 0.10). With regards to disease-free survival, ‘not-highly active’ patients—regardless of BMI groups—had an increased risk of death or recurrence [normal weight: HR: 2.58 (95% CI: 1.19–6.57), *p* = 0.06; overweight: HR: 3.10 (95% CI: 1.47–7.74), *p* = 0.01; obese: HR: 3.66 (95% CI: 1.75–9.10), *p* = 0.004]. No differences in results were observed when stratifying analyses by sex or tumor site (data not shown).

**Table 3:** Cox proportional hazard models of combined physical activity (activity threshold 18 MET hrs/wk) and BMI categories with overall survival and disease-free survival in colorectal cancer patients

	**Total N** **(N events)**	**HR (95% HR Profile Likelihood CI)**	***P*-value**	*P*-value*	**Type III** ***P*-value**
**Overall survival**					
**Highly active (≥ 18 MET hrs/wk)**					0.34
Normal weight (< 25 kg/m^2^)	82 (5)	1.00 (Ref)			
Overweight BMI: ≥ 25 and < 30 kg/m^2^)	106 (15)	2.41 (0.95–7.07)	0.08	0.15	
Obese (≥ 30 kg/m^2^)	54 (6)	1.66 (0.49–5.65)	0.41	0.65	
**Not-highly active (< 18 MET hrs/wk)**					
Normal weight (< 25 kg/m^2^)	185 (20)	1.64 (0.68–4.70)	0.31	0.52	
Overweight BMI: ≥ 25 and < 30 kg/m^2^)	247 (33)	2.25 (0.98–6.25)	0.08	0.15	
Obese (≥ 30 kg/m^2^)	257 (37)	**2.43 (1.08–6.71)**	**0.05**	**0.10**	
**Disease-free survival**					
**Highly active (≥ 18 MET hrs/wk)**					**0.04**
Normal weight (< 25 kg/m^2^)	82 (6)	1.00 (Ref)			
Overweight BMI: ≥ 25 and < 30 kg/m^2^)	106 (23)	**3.21 (1.42–8.40)**	**0.009**	**0.02**	
Obese (≥ 30 kg/m^2^)	54 (9)	2.15 (0.77–6.30)	0.15	0.28	
**Not-highly active (< 18 MET hrs/wk)**					
Normal weight (< 25 kg/m^2^)	185 (34)	**2.58 (1.19–6.57)**	**0.03**	**0.06**	
Overweight BMI: ≥ 25 and < 30 kg/m^2^)	247 (54)	**3.10 (1.47–7.74)**	**0.007**	**0.01**	
Obese (≥ 30 kg/m^2^)	257 (62)	**3.66 (1.75–9.10)**	**0.002**	**0.004**	

^*^p value corrected for multiple testing using Bonferroni correction method. All analyses were adjusted for age, sex, tumor stage, adjuvant treatment; *HR* – hazard ratio, *95% CI* – 95% confidence interval, *MET* – metabolic equivalent per task, hrs/wk – hours/week

Using 8.75 MET hrs/wk as activity threshold, ‘overweight’ and ‘obese’ patients who reported to be ‘inactive’ had an over twofold increased risk of death compared to ‘active/normal weight’ patients (*p* = 0.08 and 0.04, respectively). Similar observations were made for disease-free survival (Supplementary Table S2). A 92% increased risk of death or recurrence was observed for ‘active/overweight’ patients *vs.* ‘active/normal weight’ patients (*p* = 0.06). No differences in results were observed when stratifying analyses by sex or tumor site.

## Discussion

Here, we investigated individual and combined associations physical activity and BMI groups with overall and disease-free survival in colorectal cancer patients. Patients engaging in less than 18 MET hrs/wk, as well as ‘overweight’ and ‘obese’ patients had a 50% increased risk of death or recurrence. Notably, disease-free survival was similar for ‘overweight’ and ‘obese’ patients. When testing combined physical activity and BMI groups, ‘normal weight’ and ‘obese’ patients, but not ‘overweight’ patients, exceeding physical activity levels of 18 MET hrs/wk had improved survival outcomes compared to their ‘not-highly active’ counterparts. Smaller effect sizes were observed when using the physical activity guidelines (8.75 MET hrs/wk) as activity threshold.

Our data indicate that increased physical activity at cancer diagnosis (or pre-surgery) is associated with improved survival outcomes among colorectal cancer patients at a median follow-up of 34 months. While physical activity levels corresponding to the current guidelines for cancer prevention were not associated with survival outcomes, patients engaging in 18 or more MET hrs/wk had a 52% reduced risk of death or recurrence. A patient can achieve 18 MET hrs/wk with a variety of activities, for example, by running 90 min per week or walking 6 h per week [5]. Our results support previous research showing a 67% reduced colorectal cancer-specific mortality risk among patients participating in ≥ 18 MET hrs/wk [5, 16]. These studies have reported that this threshold is beneficial for colorectal cancer patients with particular molecular characteristics (e.g., increased *p27 *expression, activation of *CTNNBI*) [16, 17]. Our results indicate the need to conduct exercise intervention studies and cohort studies using more comprehensive physical activity measurements to further evaluate existing activity guideline thresholds for cancer survivors.

‘Overweight’ and ‘obese’ patients had worse disease-free survival compared to ‘normal weight’ patients. While the linear association between BMI and colorectal cancer risk has been clearly established, the effect of BMI on colorectal cancer survival outcomes is far more complex [24]. Research findings indicate improved survival for patients with overweight and class I obesity compared to underweight, normal weight patients, and patients with morbid obesity, although results remain inconclusive [21, 40]. A recent review concluded slightly improved survival outcomes for overweight, but worse survival outcomes for patients with obesity compared to normal weight patients [40]. Selection bias, unknown confounding, reverse causality, as well as methodological biases including BMI being limited in accurately measuring body composition have been proposed to underlie the discrepancies in the literature [21, 24, 41, 42].

Patients engaging in more than 18 MET hrs/wk had moderately improved survival outcomes compared to patients below this threshold, for both ‘normal weight’ and ‘obese’ patients. This effect was less observed when using the physical activity guidelines (8.75 MET hrs/wk) as threshold. ‘Highly active and active/obese’ patients had a similar risk of death as ‘not-highly active and inactive/normal weight’ patients and disease-free survival was improved in ‘highly active/active’ patients compared to their ‘not-highly active/inactive’ counterparts, regardless of BMI. Our results indicate that the ‘fat-but-fit’ paradox may be applicable to colorectal cancer patients. To date, the “fat-but-fit” paradox has mostly been studied in the context of cardiovascular diseases and limited evidence exists in colorectal cancer patients [8–11, 43]. A meta-analysis revealed that physically inactive individuals without cancer had a two-fold increased risk of cardiovascular-related mortality regardless of their BMI [11]. A more recent study expanded on these results showing a dose–response of physical activity on cardiovascular-related deaths across all BMI categories [43]. Physical activity may counteract the obesity-associated metabolic phenotype including systemic inflammation and insulin resistance, also referred to as ‘metabolically healthy obesity’ [12, 13]. Overall, our results suggest BMI to be a strong predictor of overall and disease-free survival among colorectal cancer patients. Physical activity, especially activity levels over 18 MET hrs/wk may counteract the negative effects of BMI on survival outcomes in this population. More large-scale studies are needed to confirm our results and expand on mechanistic insights on the potential ‘fat-but-fit’ paradox among cancer patients.

This study has several strengths and limitations. To date, this is the first prospective study in colorectal cancer patients investigating combinations of physical activity levels and BMI on colorectal cancer survival and recurrence. Medical chart abstraction, as well as linkages to cancer registry and vital status records were used to obtain detailed information on survival and recurrence outcomes. A limitation of this study is its observational nature. Although the ColoCare Study assesses a multitude of potential confounders, the possibility of residual confounding remains. Our sample size was limited resulting in large confidence intervals. Nevertheless, this is the largest study, to date, investigating this clinically pertinent research question. Cohort studies with larger sample sizes are needed to confirm our results and correctly quantify effect sizes. Larger studies will further be able to assess different effect sizes by clinicodemographic characteristics (e.g., sex, tumor site). Physical activity was self-reported, which may have introduced misclassification. Yet, misclassification due to recall bias would be assumed to be non-differential showing an effect closer to the null effect and, therefore, our results are expected to represent a smaller observed effect size compared to the actual effect size. Baseline characteristics did not differ by patients who had complete physical activity data compared to those who did not (Supplementary Table S3). Further, the IPAQ questionnaire has previously been validated using international datasets and shown acceptable measurement of moderate to vigorous activities compared to objective measurements and its validity is comparable to other self-reported assessments [32, 44]. Future studies should expand on using longitudinal assessments of physical activity to reflect changes in physical activity due to disease and treatment. BMI is limited in accurately measuring body composition. More comprehensive body composition measurements to differentiate between adipose tissue and muscle mass are needed. Improved survival with increased physical activity may be due to enhanced cardiometabolic function. Future studies are needed including disease-specific survival data.

## Conclusions

Taken together, physical activity and BMI – components of energy balance – were individually associated with colorectal cancer survival outcomes. Increased physical activity levels may counteract the negative effects of BMI on survival. The beneficial effects of physical activity were more prominent for patients engaging in more than 18 MET hrs/wk. Our data highlights the need for a more thorough analysis of physical activity-survival associations, to derive at the most meaningful recommendations in terms of survival benefit for colorectal cancer patients. Larger studies are needed to further elucidate the ‘fat-but-fit’ paradox in the context of colorectal cancer survivorship. Our study further highlights the importance to promote physical activity guidelines, and weight management throughout the cancer care continuum.

## Supplementary Information

**Additional file 1:** **Supplementary Table S1.** Cox proportional hazard models of physical activity quartiles with overall and disease-free survival in colorectal cancer patients. **Supplementary Table S2.** Cox proportional hazard models of combined physical activity (8.75 MET hrs/wk threshold) and BMI groups with overall and disease-free survival in colorectal cancer patients. **Supplementary Table S3.** Baseline study population characteristics by completed and missing physical activity data [n (%) if not otherwise stated]. **Supplementary Figure S1.** Individual and combined physical activity and BMI groups.

## Data Availability

The dataset used during the current study are available from the corresponding author on reasonable request.
