# Supplementary material for: Associations of combined physical activity and body mass index groups with colorectal cancer survival outcomes
Source: BMC Cancer. 2023 Apr 3;23:300. doi: 10.1186/s12885-023-10695-8 (PMC10069054; doi:10.1186/s12885-023-10695-8)
Supplement: Supplementary file 1 — Additional file 1: Supplementary Table S1. Cox proportional hazard models of physical activity quartiles with overall and disease-free survival in colorectal cancer patients. Supplementary Table S2. Cox proportional hazard models of combined physical activity (8.75 MET hrs/wk threshold) and BMI groups with overall and disease-free survival in colorectal cancer patients. Supplementary Table S3. Baseline study population characteristics by completed and missing physical activity data [n (%) if not otherwise stated]. Supplementary Figure S1. Individual and combined physical activity and BMI groups. [file 12885_2023_10695_MOESM1_ESM.docx]

**Supplementary Data**

**Associations of combined physical activity and body mass index groups with colorectal cancer survival outcomes and recurrence**

Himbert C^1,2^, Ose J^1,2^, Gigic B^3^, Viskochil R^1,4^, Santuci K^2^, Lin T^1,2^, Ashworth A^2^, Cohan JN^1,2^, Scaife CL^1,2^, Jedrzkiewicz J^1,2^, Damerell V^3^, Atkins KM^5^, Gong J^5^, Mutch MG^6^, Bernadt C^6^, Felder S^7^, Sanchez J^7^, Cohen SA^8^, Krane MK^8^, Hinkle N^9^, Wood E^9^, Peoples AR^1,2^, Figueiredo JC^5^, Toriola AT^6^, Siegel EM^7^, Li CI^8^, Shibata D^9^, Boucher K^1,2^, Round JL^1^, Ulrich AB^10^, Schneider M^3^, Huang LC^1,2^, Hardikar S^1,2*^, Ulrich CM^1,2*^

^1^ University of Utah, Salt Lake City, UT

^2^ Huntsman Cancer Institute, Salt Lake City, UT

^3^ Heidelberg University Hospital, Germany

^4^ University of Massachusetts Boston, Boston, MA

^5^ Cedars-Sinai Medical Center, Los Angeles, CA

^6^ Washington University School of Medicine, St. Louis, MO

^7^ H. Lee Moffitt Cancer Center & Research Institute, Tampa, FL

^8^ Fred Hutchinson Cancer Center and University of Washington, Seattle, WA

^9^ University of Tennessee Health Science Center, Memphis, TN

^10^ Rheinland Klinikum Neuss LukasKrankenhaus, Germany

*These authors contributed equally.

| **Supplementary Table S1. Cox proportional hazard models of physical activity quartiles with overall and disease-free survival in colorectal cancer patients** | | | | |
| --- | --- | --- | --- | --- |
|  | | **Adjusted Model*** | | |
|  | **MET hrs/wk**  **mean (range)** | **HR (95% CI)** | **P-value** | **p for trend** |
| **Overall survival** | | | | |
| Quartile 1 | 0.99 (0.97-1.00) | 1.00 (Ref) | | **0.01** |
| Quartile 2 | 3.97 (1.46-6.56) | 0.73 (0.45-1.21) | 0.23 |  |
| Quartile 3 | 11.6 (3.38-6.83) | **0.58 (0.35-0.96)** | **0.03** |  |
| Quartile 4 | 36.7 (18.5-97.9) | **0.55 (0.33-0.92)** | **0.02** |  |
| **Disease-free survival** | | | | |
| Quartile 1 | 0.99 (0.97-1.00) | 1.00 (Ref) | | **0.04** |
| Quartile 2 | 3.97 (1.46-6.56) | 0.70 (0.47-1.04) | 0.08 |  |
| Quartile 3 | 11.6 (3.38-6.83) | 0.81 (0.57-1.17) | 0.26 |  |
| Quartile 4 | 36.7 (18.5-97.9) | **0.51 (0.34-0.77)** | **0.001** |  |
| *adjusted for age, sex, stage at diagnosis, adjuvant treatment; HR – Hazard Ratio, 95% CI – 95% Confidence Interval, MET – metabolic equivalent per task, hrs/wk – hours/week | | | | |

| **Supplementary Table S2. Cox proportional hazard models of combined physical activity (8.75 MET hrs/wk threshold) and BMI groups with overall and disease-free survival in colorectal cancer patients** | | | | | |
| --- | --- | --- | --- | --- | --- |
|  | **Total N**  **(N events)** | **HR (95% HR Profile Likelihood CI)** | **P-value** | **P-value*** | **Type III**  **P-value** |
| **Overall survival** | | | | | |
| **Active (≥8.75 MET hrs/wk)** | | | | | 0.30 |
| Normal weight (<25 kg/m^2^) | 142 (10) | 1.00 (Ref) | | |  |
| Overweight (≥25 and <30 kg/m^2^) | 166 (22) | **2.22 (1.09-4.85)** | **0.04** | **0.08** |  |
| Obese (≥30 kg/m^2^) | 99 (13) | 2.06 (0.90-4.80) | 0.09 | 0.17 |  |
| **Inactive (<8.75 MET hrs/wk)** | | | | |  |
| Normal weight (<25 kg/m^2^) | 125 (15) | 1.96 (0.89-4.46) | 0.10 | 0.19 |  |
| Overweight BMI: ≥25 and <30 kg/m^2^) | 187 (26) | **2.25 (1.09-4.85)** | **0.04** | **0.08** |  |
| Obese (≥30 kg/m^2^) | 212 (30) | **2.31 (1.18-4.89)** | **0.02** | **0.04** |  |
| **Disease-free survival** | | | | | |
| **Active (≥8.75 MET hrs/wk)** | | | | |  |
| Normal weight (<25 kg/m^2^) | 142 (17) | 1.00 (Ref) | | | 0.12 |
| Overweight (≥25 and <30 kg/m^2^) | 166 (35) | **1.92 (1.09-3.49)** | **0.03** | **0.06** |  |
| Obese (≥30 kg/m^2^) | 99 (22) | **1.96 (1.04-3.72)** | **0.04** | **0.08** |  |
| **Inactive (<8.75 MET hrs/wk)** | | | | |  |
| Normal weight (<25 kg/m^2^) | 125 (23) | 1.67 (0.90-3.20) | 0.10 | 0.19 |  |
| Overweight (≥25 and <30 kg/m^2^) | 187 (42) | **2.03 (1.18-3.64)** | **0.02** | **0.04** |  |
| Obese (≥30 kg/m^2^) | 212 (49) | **2.22 (1.31-3.94)** | **0.005** | **0.01** |  |
| *p value corrected for multiple testing using Bonferroni correction method. ^a^adjusted for age, sex, stage at diagnosis, adjuvant treatment; HR – Hazard Ratio, 95% CI – 95% Confidence Interval, MET – metabolic equivalent per task, hrs/wk – hours/week | | | | | |

| **Supplementary Table S3. Baseline study population characteristics by completed and missing physical activity data** [n (%) if not otherwise stated] | | |
| --- | --- | --- |
|  | **Physical activity data missing** | **Physical activity data complete** |
| **N** | 392 | 931 |
| **Age, mean (SD)** | 62 (±11) | 60 (±13) |
| **Sex** | | |
| Female | 168 (43) | 405 (43) |
| Male | 224 (57) | 526 (57) |
| **Race** | | |
| White | 288 (73) | 815 (87) |
| Non-White | 92 (23) | 110 (12) |
| **Ethnicity** | | |
| Hispanic | 25 (7) | 29 (3) |
| Non-Hispanic | 345 (93) | 902 (97) |
| **Tumor Stage** | | |
| I | 71 (20) | 216 (23) |
| II | 121 (33) | 256 (28) |
| III | 171 (47) | 453 (49) |
| **Tumor site** | | |
| Colon | 199 (54) | 478 (51) |
| Rectum | 169 (46) | 453 (49) |
| **Neoadjuvant treatment** | | |
| Yes | 116 (32) | 279 (30) |
| No | 244 (68) | 652 (70) |
| **Adjuvant treatment** | | |
| Yes | 171 (47) | 401 (43) |
| No | 196 (53) | 530 (57) |
| **Vital status** | | |
| Alive | 332 (85) | 815 (87) |
| Deceased | 60 (15) | 116 (13) |
| **Recurrence** | | |
| Yes | 78 (22) | 153 (17) |
| No | 275 (78) | 751 (83) |
| SD – standard deviation, MET hrs/wk – metabolic equivalent per task in hours per week, BMI – body mass index | | |
|  | | |

**
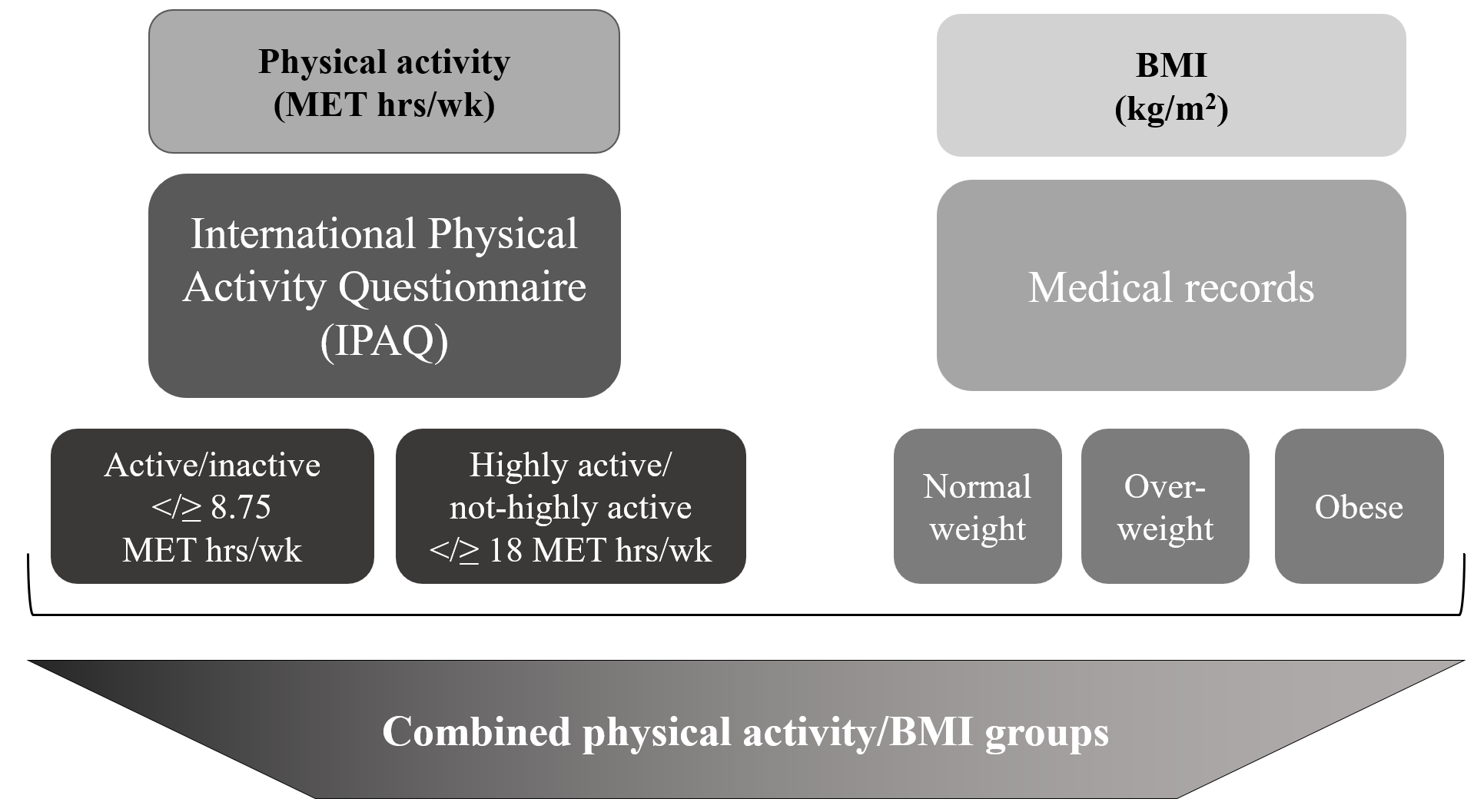
**

**Supplementary Figure S1.** Individual and combined physical activity and BMI groups.
